# Cleavage of 3′-terminal adenosine by archaeal ATP-dependent RNA ligase

**DOI:** 10.1038/s41598-017-11693-0

**Published:** 2017-09-14

**Authors:** Shigeo Yoshinari, Yancheng Liu, Paul Gollnick, C. Kiong Ho

**Affiliations:** 1 Department of Biological Sciences, State University of New York, Buffalo, NY 14260 United States of America; 2 Human Biology Program, School of Integrative and Global Majors, University of Tsukuba, Ibaraki, 305-8575 Japan; 3 Department of Infection Biology, Faculty of Medicine, University of Tsukuba, Ibaraki, 305-8575 Japan

## Abstract

*Methanothermobacter thermoautotrophicus* RNA ligase (MthRnl) catalyzes formation of phosphodiester bonds between the 5′-phosphate and 3′-hydroxyl termini of single-stranded RNAs. It can also react with RNA with a 3′-phosphate end to generate a 2′,3′-cyclic phosphate. Here, we show that MthRnl can additionally remove adenosine from the 3′-terminus of the RNA to produce 3′-deadenylated RNA, RNA(3′-rA). This 3′-deadenylation activity is metal-dependent and requires a 2′-hydroxyl at both the terminal adenosine and the penultimate nucleoside. Residues that contact the ATP/AMP in the MthRnl crystal structures are essential for the 3′-deadenylation activity, suggesting that 3′-adenosine may occupy the ATP-binding pocket. The 3′-end of cleaved RNA(3′-rA) consists of 2′,3′-cyclic phosphate which protects RNA(3′-rA) from ligation and further deadenylation. These findings suggest that ATP-dependent RNA ligase may act on a specific set of 3′-adenylated RNAs to regulate their processing and downstream biological events.

## Introduction

2′,3′-cyclic phosphates at the 3′-termini of RNAs play important roles in RNA metabolism. This cyclic phosphate and a 5′-OH end are formed by a transesterification reaction, in which the 2′-OH on the ribose attacks the adjacent 3′-phosphate, breaking the phosphodiester backbone of the RNA, during either chemical or enzymatic cleavage^1^. A variety of ribonucleases can catalyze this step, including: the tRNA splicing endonuclease, which removes introns in tRNAs^2^; Ire1 endonuclease, which cleaves the *HAC1* mRNA during the unfolded protein response^3,4^; Usb1/Msp1 3′-5′ exoribonuclease, which trims the oligouridine tail for maturation of the U6 snRNA^5–8^; and type 1 DNA topoisomerase, which can cleave RNA through formation of covalent topoisomerase–RNA intermediate^9^. The 2′,3′-cyclic phosphate end can also be generated by conversion of an RNA terminating in a 3′-PO_4_, by the RNA cyclase RtcA^10–12^ and a number of thermophilic polynucleotide ligases^13^. This reaction involves transfer of AMP from ATP to the RNA 3′-phosphate to form an RNA(3′)pp(5′)A intermediate, which is subsequently attacked by the 2′-OH to yield cyclic ends, a reaction that liberates AMP.

Interest in the 2′,3′-cyclic phosphate resurged following identification of a GTP-dependent RNA ligase, RtcB, that acts specifically on cyclic ends. The 2′,3′-cyclic phosphate and 5′-OH terminus generated during bacterial RNA repair, mammalian and archaea tRNA splicing, and metazoan unfolded protein response are joined to form a phosphodiester bond by RtcB^14–22^. RtcB reacts with GTP to form RtcB-GMP, converts the 2′,3′-cyclic phosphate into 3′-PO_4,_ transfers the GMP to the 3′-PO_4_ end to form an RNAp(pG) intermediate^23–25^. It then forms a phosphodiester linkage by nucleophilic attack, with 5′-OH liberating GMP. ATP-dependent RNA ligation involves a series of nucleotidyl transfer steps similar to those that occur during ligation via the RtcB pathway, with the ligase reacting with ATP to form a covalent ligase-AMP, followed by transfer of the AMP to the 5′-PO_4_ end of the RNA to form an AppRNA intermediate, and subsequent formation of a phosphodiester bond by nucleophilic attack, with 3′-OH releasing AMP^26–28^.

Several archaea species encode both GTP-dependent and ATP-dependent RNA ligases. *Methanothermobacter thermoautotrophicus* RNA ligase (MthRnl) is an ATP-dependent RNA ligase that belongs to the Rnl3 family and catalyzes the circularization of single-stranded RNA, as well as DNA^29^. Under non-optimal conditions, MthRnl can remove 5′-AMP from the AppRNA intermediate to generate a 5′-PO_4_ RNA^29,30^. MthRnl can also transfer AMP to RNA containing 3′-PO_4_ termini to form 2′,3′-cyclic phosphate^13^. While there is growing interest for the use of thermostable RNA ligases in constructing sequencing libraries of small RNAs, including microRNAs, its physiological/optimal polynucleotide substrate is not known. In this report, we show that MthRnl cleaves the adenosine residue from the 3′-OH end and leaves RNAs with 2′,3′-cyclic phosphates. Notably, cleavage is selective, with only a single adenosine residue removed. These findings raise the possibility that the RNA ligase acts as a surveillance/editing enzyme that selectively converts the reactive 3′-OH into a 2′,3′-cyclic phosphate end, thereby regulating their processing and downstream biological events.

## Results

### MthRn1 generates a novel RNA

**Figure 1:**
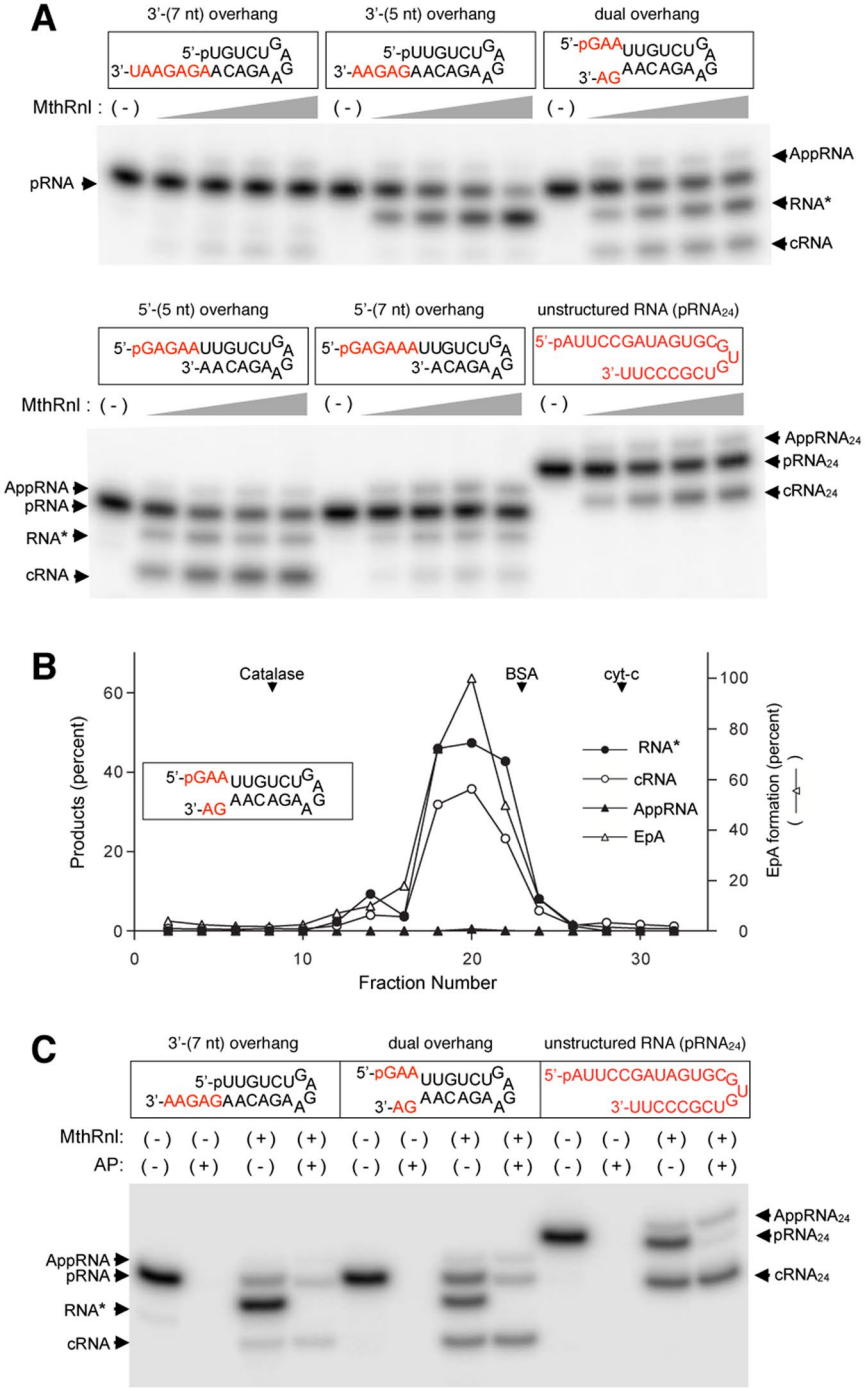
MthRn1 activity﻿ generates a novel RNA*. (**A**) Effectiveness of ligation of pRNA substrates with various 5′- and 3′-overhangs, as assessed by ligation assay. Ligations were performed in 20 µl containing 1 pmol of indicated^32^P-labeled pRNA and 0.23, 0.45, 0.90 or 1.8 µg of MthRnl (from left to right in each titration series). Reaction without MthRnl (-). Positions of input pRNA, 5′ adenylated RNA (AppRNA), and circularized RNA (cRNA) are indicated. A novel RNA product that migrates between pRNA and AppRNA is denoted by asterisk (RNA*). (**B**) Sedimentation analysis of MthRnl activities.  MthRnl (100 µg) was centrifuged for 20 hrs at 4 °C in a 15–30% glycerol gradient as described previously^29^. The amount of ligation product formed was gauged by signal intensity as a proportion of total RNA product (y-axis). The amount of EpA formed was gauged by the signal intensity of radiolabeled MthRnl (yy-axis). The peaks of the marker proteins, catalase, BSA and cytochrome c, are indicated. (**C**) RNA* has an exposed 5′-phosphate. Standard ligation reaction mixtures (20 µl) containing 0.45 µg of MthRnl and 1 pmol of the indicated pRNA was incubated at 55 °C for 15 min. Reaction was terminated by addition of EDTA, after which the samples were incubated with or without 0.2 unit of alkaline phosphatase (AP) at 37 °C for 30 min. Sample without enzyme served as negative control (-). Positions of pRNA substrate, AppRNA, cRNA and RNA* are indicated.

Previously MthRnl was shown to circularize a 24-mer unstructured single-stranded pRNA that migrates 2-nts faster than the input linear substrate on a high percent PAGE gel^29^ (Fig. 1A; unstructured 24-mer pRNA). Toward identifying the optimal substrate for MthRnl ligation activity, we prepared five different ^32^P-labeled 21-mer pRNA substrates that mimic hairpin structured RNAs that are expected to form overhangs at the 5′- and/or 3′-end (Fig. 1A) Incubation of recombinant MthRnl with the 3′-(5nt) overhang, dual overhang, 5′-(5nt) overhang or 5′-(7nt) overhang pRNA resulted in the generation of a novel RNA species (RNA*) that migrates between the input linear pRNA and cRNA (Fig. 1A). In the case of the 3′-(5nt) overhang RNA, the majority was converted to RNA*. In the case of the dual overhang RNA, the ratio of RNA*:cRNA formed was 2:1, and with the 5′-(5nt) overhang RNA, it was 1:3. The 5′-(7nt) overhang RNA was a poor substrate for formation of both RNA* and cRNA. Moreover, the 3′-(7nt) overhang RNA did not circularized as efficiently as the other RNA substrates tested. As discussed below, RNA* is only detected from RNAs that contain adenine at the 3′-terminus. Neither the 3′-(7nt) overhang pRNA substrate nor the unstructured 24-mer pRNA, each of which has uracil at the 3′-terminus, were converted to RNA*.

To verify that the observed RNA* was a product of MthRnl activity and not due to bacterial contamination, we subjected recombinant MthRnl to glycerol sedimentation analysis. The RNA* forming activity co-sedimented with the adenylatranferase, RNA-adenylate and RNA circularization activities (Fig. 1B), suggesting that MthRnl is responsible for generating RNA*. Neither T4 RNA Ligase 1 (Rnl1)^31^ nor T4 RNA Ligase 2 (Rnl2)^32^ was capable of forming RNA* from any of the RNA substrates tested (Supplementary Fig. S1). We also cloned and purified the MthRnl homolog *Thermococcus kodakarensis* RNA ligase (TkoRnl), and demonstrated that the recombinant protein is likewise capable of generating RNA*, specifically from RNA with a 3′-adenine (Supplementary Fig. S2). Thus, the formation of RNA* is likely conserved in archaeal ATP-dependent RNA ligases. The 3′-(5nt) overhang and the dual overhang pRNA substrates were used to further characterize the RNA* generating activity.

### MthRnl removes adenosine from 3′-end of the RNA

We hypothesized that RNA* is either a linear pRNA with a modification at the 3′-end or a closed circular RNA that migrates differently from a conventional cRNA in the gel. To distinguish between these two possibilities, reaction products generated by MthRnl were treated with an alkaline phosphatase. The RNA*s generated from both the 3′-(5nt) overhang and dual overhang pRNA substrates were sensitive to alkaline phosphatase treatment, indicating that the 5′-radiolabeled phosphate on RNA* is exposed (Fig. 1C). These results imply that RNA* is generated by removal of a single nucleoside from the 3′-end of pRNA.

**Figure 2:**
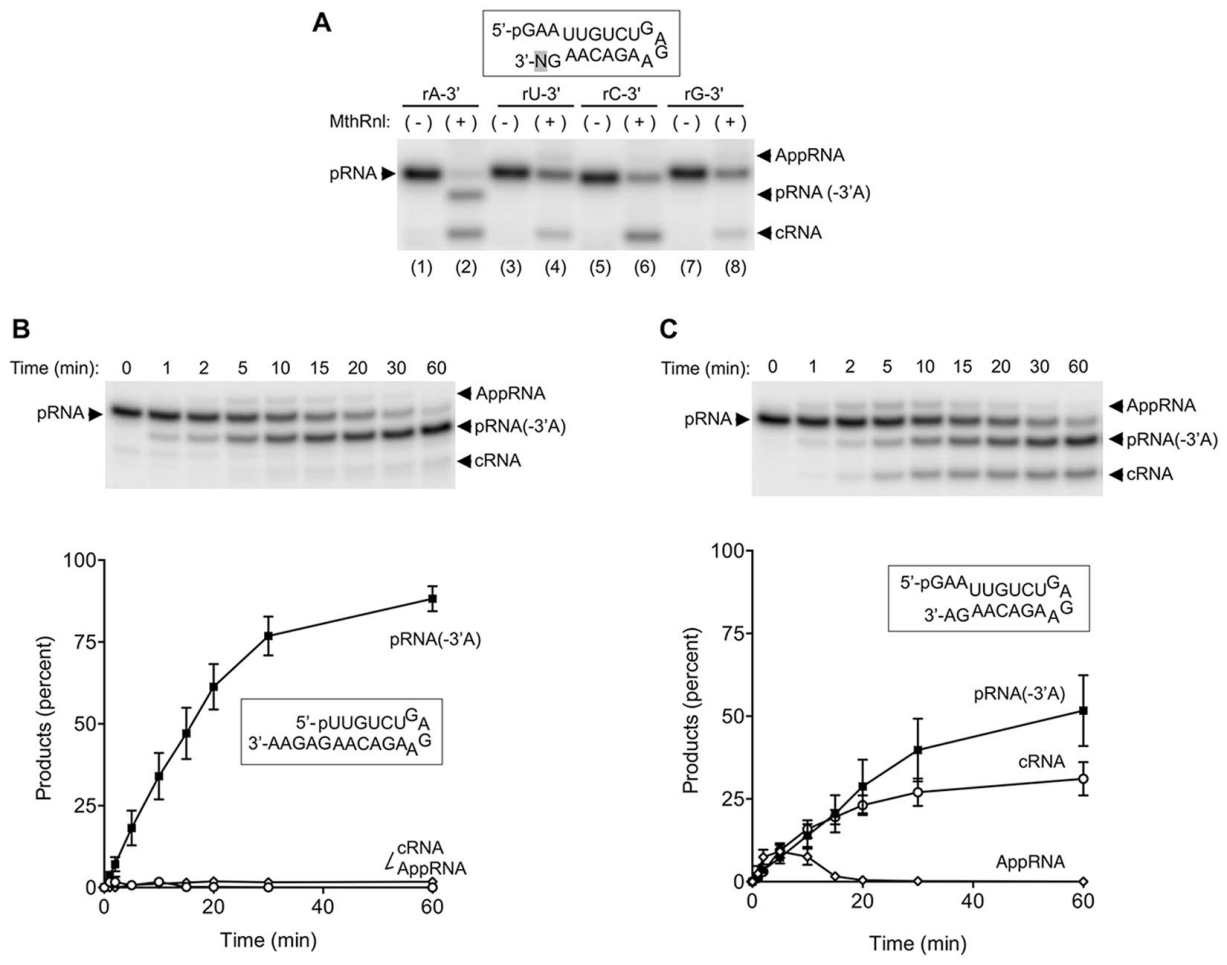
MthRnl cleaves adenosine from 3′-end of the RNA. (**A**) Specificity for adenine at the 3′-end. Standard ligation assay (20 µl) contained 0.9 µg of MthRnl and 1 pmol of dual overhang pRNA with either rA, rU, rC or rG at the 3′-terminus. The position of the variant nucleotide (N) is shaded in gray in the structures of dual overhang pRNA substrates. Positions of input pRNA, AppRNA, cRNA and 3′-deadenylated RNA [pRNA(-3′A)] are indicated next the gel. (**B**) Kinetic analysis. A reaction mixture (100 µl) containing 50 mM Tris-HCl (pH 6.5), 0.5 mM MgCl_2_, 10 pmol 3′-(5nt) over-hang pRNA and 2.25 µg of MthRnl was incubated at 55 °C. Aliquots (10 µl) were withdrawn at the times indicated and quenched immediately with formamide-EDTA. Top: A representative gel from one experiment. Bottom: The fraction of deadenylated RNA [pRNA(-3′A)], AppRNA, and cRNA product is plotted as a function of incubation time. (**C**) Identical to (**B**) except that the substrate for ligation was dual-overhang pRNA as indicated. The data shown represent the average of three separate experiments with SE bars.

As described above, RNA* was observed on pRNA substrates that contain adenine, but not a uracil at the 3′-terminus (Fig. 1A). This specificity was further tested using dual overhang RNA substrates in which the 3′-terminal adenine is replaced with uracil, cytosine or guanine (Fig. 2A). RNA* was detected only when a pRNA substrate containing a 3′-terminal adenosine was used, implying that MthRnl removes specifically 3′-adenosine. Thus, henceforth we refer to RNA* as pRNA(-3′A).

### The 3′-deadenylation activity is independent from RNA ligation activity

Kinetic analysis revealed that the majority of the input 3′-(5nt) overhang pRNA is converted to pRNA(-3′A) in 60 min (Fig. 2B). The initial rate of 3′-deadenylation is ~10 fmol/min of 3′-(5nt) overhang pRNA per pmol of enzyme, which is comparable to the initial rate of RNA circularization; 16 fmol/min of pRNA circularization per pmol of enzyme^30^. The 3′-deadenylation activity with 3′-(5nt) overhang pRNA substrate was ~ 2-fold higher than that with the dual overhang pRNA substrate (Fig. 2C). In the case of the dual overhang pRNA substrate, approximately 50% was converted to pRNA(-3′A) in 60 min, whereas the remaining substrate was converted to an AppRNA at an early time point and subsequently to cRNA. Because pRNA(-3′A) is generated independent of the product of the ligation pathway, pRNA(-3′A) is not likely to be an intermediate of the ligation reaction.

**Figure 3:**
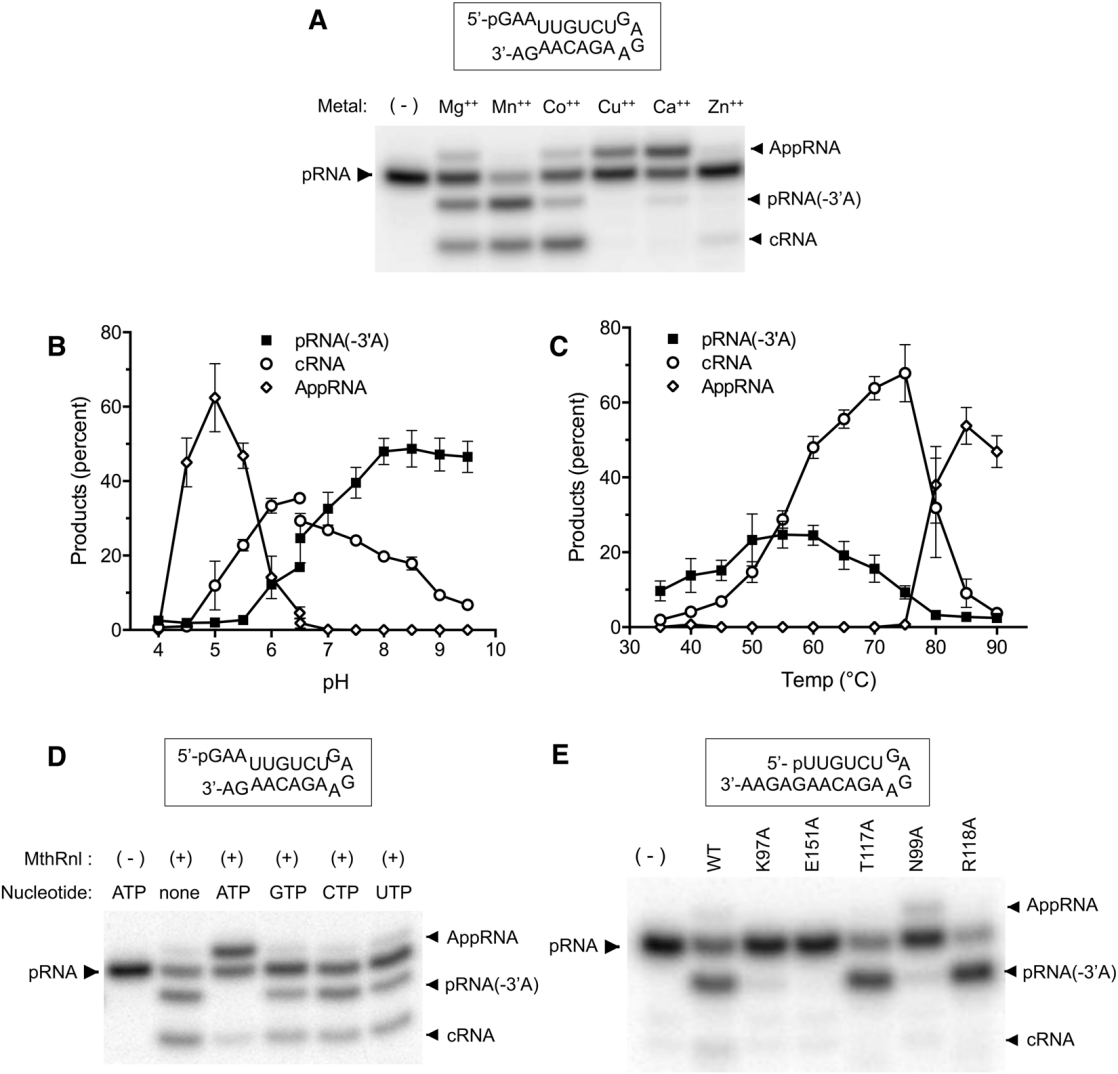
Characterization of MthRnl RNA 3′-deadenylation activity. (**A**) Specificity for divalent cations. Standard ligation reaction mixture containing 1 pmol of the indicated dual overhang pRNA substrate and 0.45 µg of MthRnl with 0.5 mM of the indicated divalent cation (chloride salt). MthRnl was omitted from a control reaction (-). Position of input pRNA, AppRNA, cRNA and 3′-deadenylated RNA [pRNA(-3′A)] are indicated next to the gel. (**B**) Effects of pH on deadenlyation activity. Standard ligation reaction mixtures containing 50 mM buffer (either Tris-Acetate between pH 4.0–6.5; or Tris-HCl between pH 6.5–9.5) and 1 pmol of the indicated dual overhang pRNA substrate and 0.45 µg of MthRnl. Level of cRNA (open circle), AppRNA (open diamond) and pRNA (-3′A) (closed square), plotted as functions of pH. The data shown represent the average of three separate experiments with SE bars. (**C**) Temperature dependence. Standard ligation reaction mixture containing indicated dual overhang pRNA substrate and MthRnl were incubated for 15 min at indicated temperature. Levels of cRNA (open circle), AppRNA (open diamond) and pRNA(-3′A) (closed square) are plotted as function of temperature. The data shown represent the average of three separate experiments with SE bars. (**D**) Effects of nucleotide triphosphates. Standard ligation reaction mixture (20 µl) containing 1 pmol of dual overhang pRNA shown in (**A**), 0.45 µg of MthRnl, and 0.2 mM rATP, rGTP, rCTP, rUTP or no NTP (none). MthRnl was omitted from a control reaction (-). (**E**) Mutation analysis. Standard ligation reaction mixture (20 µl) containing 1 pmol of the indicated 3′-(5nt) overhang pRNA substrate and 1.8 µg of wild-type (WT) and indicated mutant MthRnl protein.

Like the ligation reaction, the 3′-deadenylation activity requires a divalent cation. This requirement was satisfied by magnesium, manganese, cobalt or, to a lesser extent, calcium (Fig. 3A). MthRnl preferentially deadenylated pRNA under alkaline conditions (Fig. 3B). At pH values between 6.5 and 7.0, the dual overhang pRNA substrate was converted to pRNA(-3′A) or cRNA with similar frequency. With increasing pH, the yield of pRNA(-3′A) increased and cRNA levels declined; pRNA(-3′A) was not detectable under pH 5.5. MthRnl can deadenylate substrates over wide range of temperatures, the optimum being 55 °C. This differs from the ligation reaction, which was optimal above 55 °C (Fig. 3C). The level of pRNA(-3′A) declined steadily as the temperature increased above 55 °C, with the formation of cRNA favored. The two activities displayed distinct temperature and pH optima, implying that the ligation and 3′-deadenylation reactions are independent of one another. Nonetheless, the ATP binding site of MthRnl appears to be essential for 3′-deadenylation. Addition of ATP inhibited the MthRnl 3′-deadenylation activity, similar to the ATP inhibitory effect for the ligation activity (Fig. 3D and Supplementary Figs S1 and S3). An ATP analog, AMPPNP, which can occupy the ATP binding site, also inhibits 3′-deadenylation. In contrast, AMP or other rNTPs did not significantly affect the formation of 3′-deadeylated or circular RNA. Mutant forms of the enzyme that fail to generate EpA (K97A and E151A) or whose ligation activity is reduced (N99A) were defective for the 3′-deadenylation activity (Fig. 3E and Supplementary Fig. S4)^30^; only trace amounts of pRNA(-3′A) were detected in the presence of the K97A and N99A mutant forms of the enzyme. On the other hand, MthRnl variants that catalyze EpA formation but are defective for the step 2 or step 3 ligation reaction (T117A and R118A) were capable of forming pRNA(-3′A). We speculate that adenosine at the 3′-end of the RNA is recognized by the active site that recognizes the ATP in step 1 of the ligation reaction.

### Substrate specificity of 3′-RNA deadenylation

**Figure 4:**
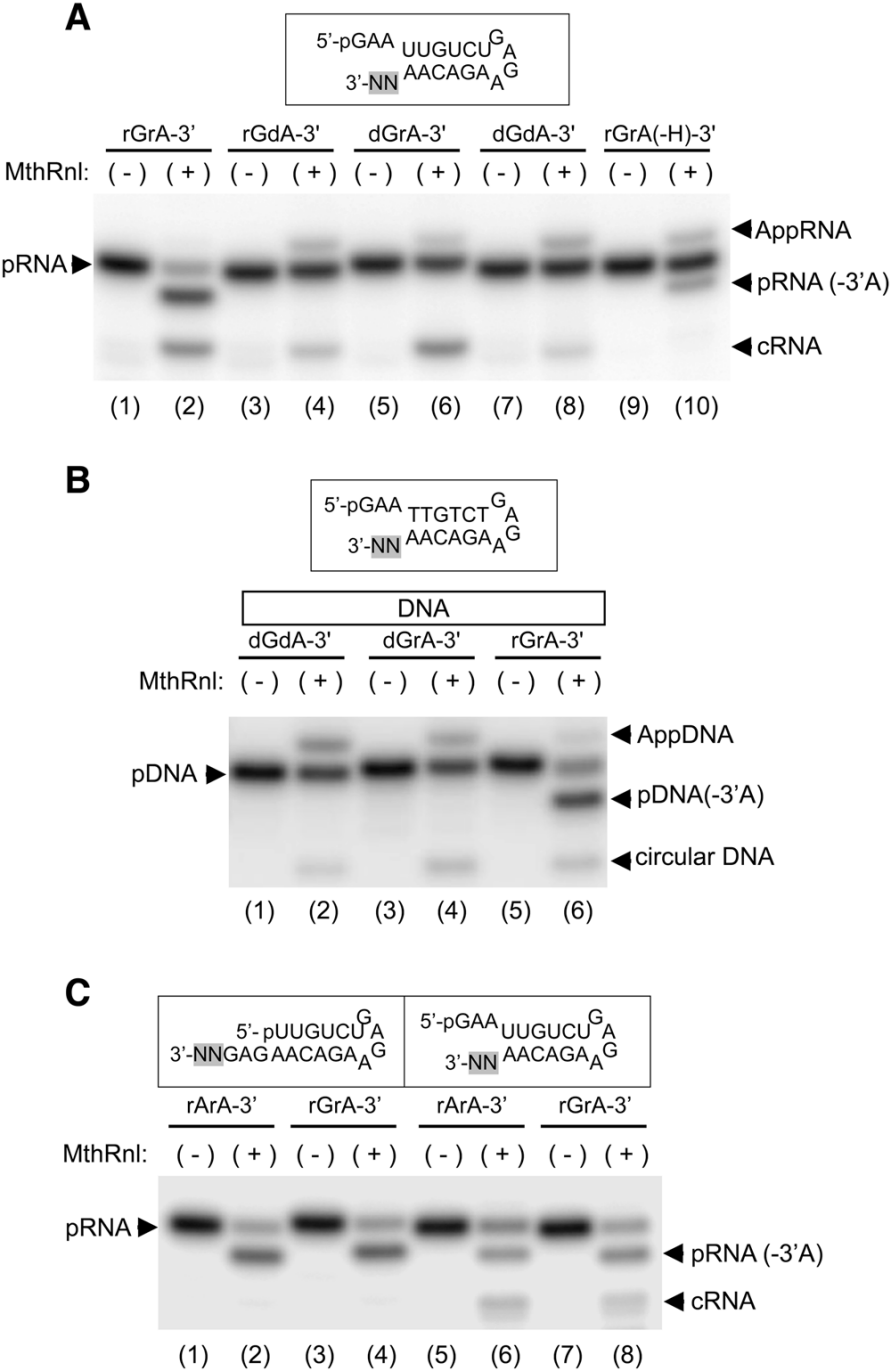
RNA requirements for 3′-deadenylation activity. (**A**) Requirements of ribonucleotide at 3′-end. The standard ligation assay (20 µl) containing 0.9 µg of MthRnl and 1 pmol of dual overhang pRNA with either 2′ deoxyribose at the 3′-end (rGdA-3′), 2′ deoxyribose at the penultimate position (dGrA-3′), two consecutive 2′ deoxyriboses at the 3′-end (dGdA-3′) or cordycepin at the 3′-end [rGrA(-H)-3′]. Control reaction with dual over-hang pRNA containing all ribose are indicated as rGrA-3′. (**B**) Effect of DNA in the strand. The standard ligation assay (20 µl) containing 0.9 µg of MthRnl and 1 pmol of either dual overhang pDNA strand (dGdA-3′), single ribose (dGrA-3′) or two consecutive ribose at 3′-end (rGrA-3′). (**C**) 3′-deadenylation is not processive. The standard ligation assay (20 µl) contained 0.9 µg of MthRnl, with either 1 pmol of the indicated pRNA containing consecutive adenosine (rArA-3′) or guanosine at penultimate position (rGrA-3′) at the 3′-end. Sample without enzyme treatment served as negative control (-). The structures of ^32^P-labeled pRNA or pDNA substrates and the positions of variant nucleotides (N) are shaded in gray. Positions of pRNA, pDNA, AppRNA, AppDNA, cRNA, circular DNA, pRNA (-3′A) and pDNA (-3′A) on the gels are indicated.

We previously showed that MthRnl can circularize single-stranded DNA^29^. To determine whether MthRnl is additionally capable of removing deoxyadenosine, we prepared RNA substrates in which the 3′-terminus was replaced with 2′-deoxyadenosine (Fig. 4A; rGdA-3′) and cordycepin (Fig. 4A; rGrA(-H)-3′). MthRnl did not remove 2′-deoxyadenosine (Fig. 4A, lane 4 and lane 8), but did remove cordycepin, albeit less efficiently than from an RNA that contains ribose at the 3′-end (Fig. 4A, lane 10). We also found that MthRnl was unable to deadenylate the substrate when the penultimate nucleotide was replaced with a deoxynuclotide (dGrA-3′; Fig. 4A, lane 6). We next prepared additional 21-mer DNAs; in one, the sequence was identical to that of the dual overhang RNA substrate (Fig. 4B: dGdA-3′); in two others, a DNA/RNA hybrid substrate consisted of either 20 deoxynucleotides with a single adenosine ribonucleotide at the 3′-end (Fig. 4B: dGrA-3′) or 19 deoxynucleotides with 2 ribonucleotides at the 3′-end (Fig. 4B: rGrA-3′). MthRnl could only deadenylate substrates in which the penultimate nucleotide is a ribonucleotide. These results imply that the 3′-deadenylation activity requires 2′-OH at both the terminal adenosine and the penultimate nucleotide, but that the 3′-OH on the terminal adenosine is not strictly required for the reaction chemistry.

The fact that MthRnl removes specifically 3′-rA raises the question of whether it can processively deadenylate RNA containing multiple adenosines. We reasoned that if MthRnl is capable of removing two consecutive adenosines, the product should migrate faster than pRNA(-3′A). However, this was not the case. The 3′-(5nt) overhang RNA substrate with two consecutive 3′-adenosines did not generate a shorter 3′-deadenylated species (Fig. 1A). To confirm this finding, we replaced guanosine at the penultimate position, with adenosine in the 3′-(5nt) overhang substrate (Fig. 4C: rGrA-3′, lane 4) and the dual overhang substrate that contains two consecutive adenosines at the 3′-end (Fig. 4C: rArA-3′, lane 2). No difference in the mobility of the 3′-deadenylated product was detected. We therefore conclude that MthRnl can cleave only a single adenosine residue from the 3′-end and, once the adenosine is cleaved, the pRNA(-3′A) cannot be ligated to form the cRNA.

### MthRnl leaves a phosphate group at the 3′-end of deadenylated RNA

**Figure 5:**
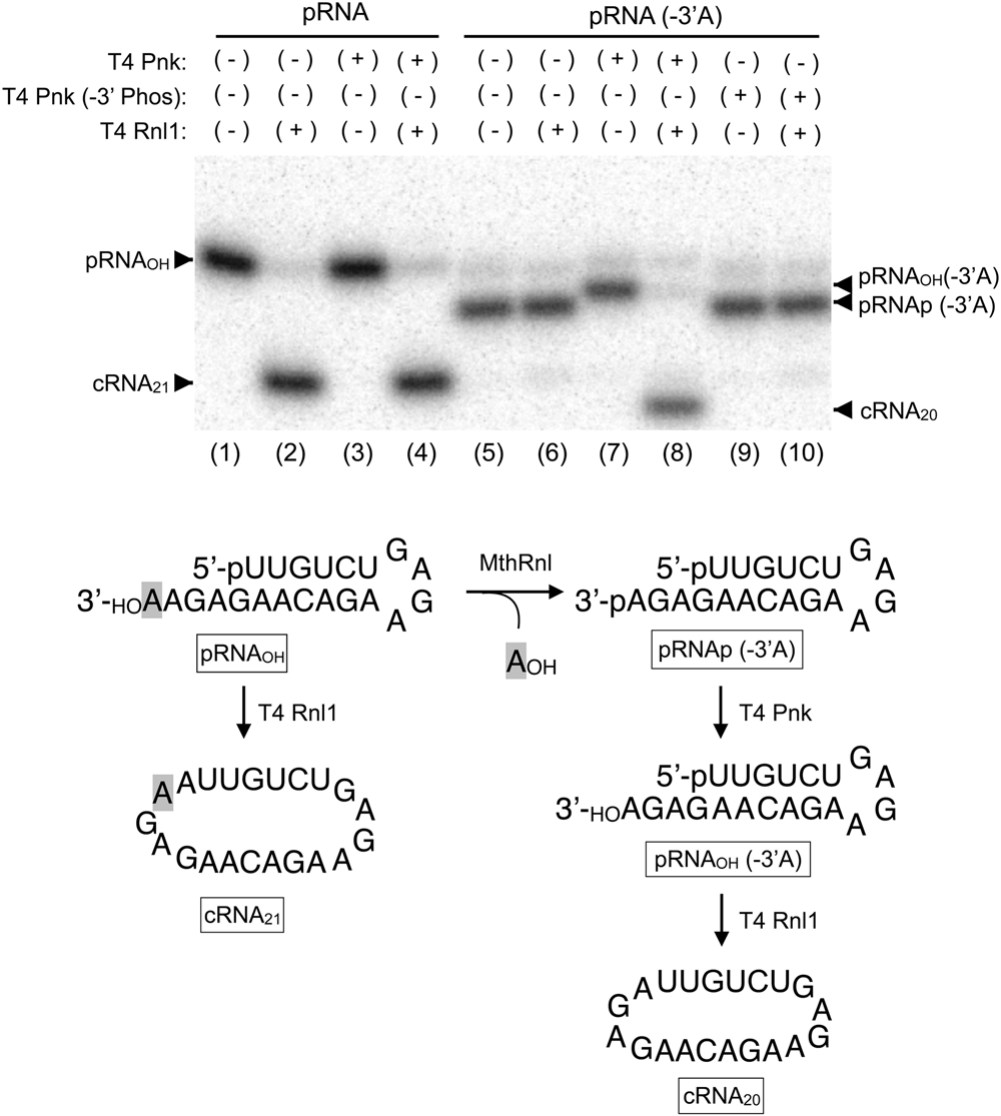
The phosphate group is retained at the 3′-end of deadenylated RNA. *Top*: PAGE Analysis. Reaction mixture (20 μl) containing 50 mM Tris-HCl (pH8.0), 2 mM DTT, 10 mM MgCl_2_, 0.2 mM ATP, 1 pmole of pRNA (-3′A) (Methods) was incubated with T4 Rnl1 (0.45 µg, lane 6), T4 Pnk (10 units, lane 7), T4 Rnl1 plus T4 Pnk (lane 8), T4 Pnk (-3′ Phos) (10 unit, lane 9), or T4 Rnl1 + T4 Pnk (-3′ Phos). Position of pRNA(-3′A) is shown in lane 5. Lanes 1 through 4 represent a control reaction using 3′-(5nt) overhang pRNA substrate without enzyme (lane 1), with T4 Rnl1 (lane 2), with T4 Pnk (lane 3), and with T4 Pnk plus T4 Rnl1 (lane 4). Bottom: Schematic diagram of reaction products.

The above-described results suggest that pRNA(-3′A) lacks a reactive 3′-OH that is necessary for the strand-joining reaction. We hypothesize that the adenosine is released and the phosphate group is retained on the 3′-end of pRNA(-3′A) by MthRnl. To test this hypothesis, we purified pRNA(-3′A) generated by MthRnl from the 3′-(5nt) overhang substrate and incubated it with T4 polynucleotide kinase (Pnk), which can convert 2′,3′-cyclic phosphate or 3′-PO_4_ into 3′-OH (Fig. 5, lane 5). Treatment with﻿ T4 Pnk shifts the 5′-labeled pRNA(-3′A) to a more slowly migrating species (Fig. 5, lane 7). This shift is likely due to a loss of negatively charged phosphate because incubation of pRNA(-3′A) with the phosphatase-deficient mutant form of T4 Pnk (-3′Phos) did not alter substrate mobility (Fig. 5; lane 9). When pRNA(-3′A) was incubated with both T4 Rnl1 and T4 Pnk, pRNA(-3′A) was converted into a 20-mer cRNA (Fig. 5; lane 8). In contrast, incubation with the mutant form of T4 Pnk (Fig. 5; lane 10) or T4 RNA ligase 1 alone (Fig. 5; lane 6) did not alter the pRNA(-3′A). These results imply that the phosphate group is retained at the 3′-end of pRNA(-3′A).

### MthRnl does not require the 5′-phosphate for the RNA 3′-deadenylation reaction

**Figure 6:**
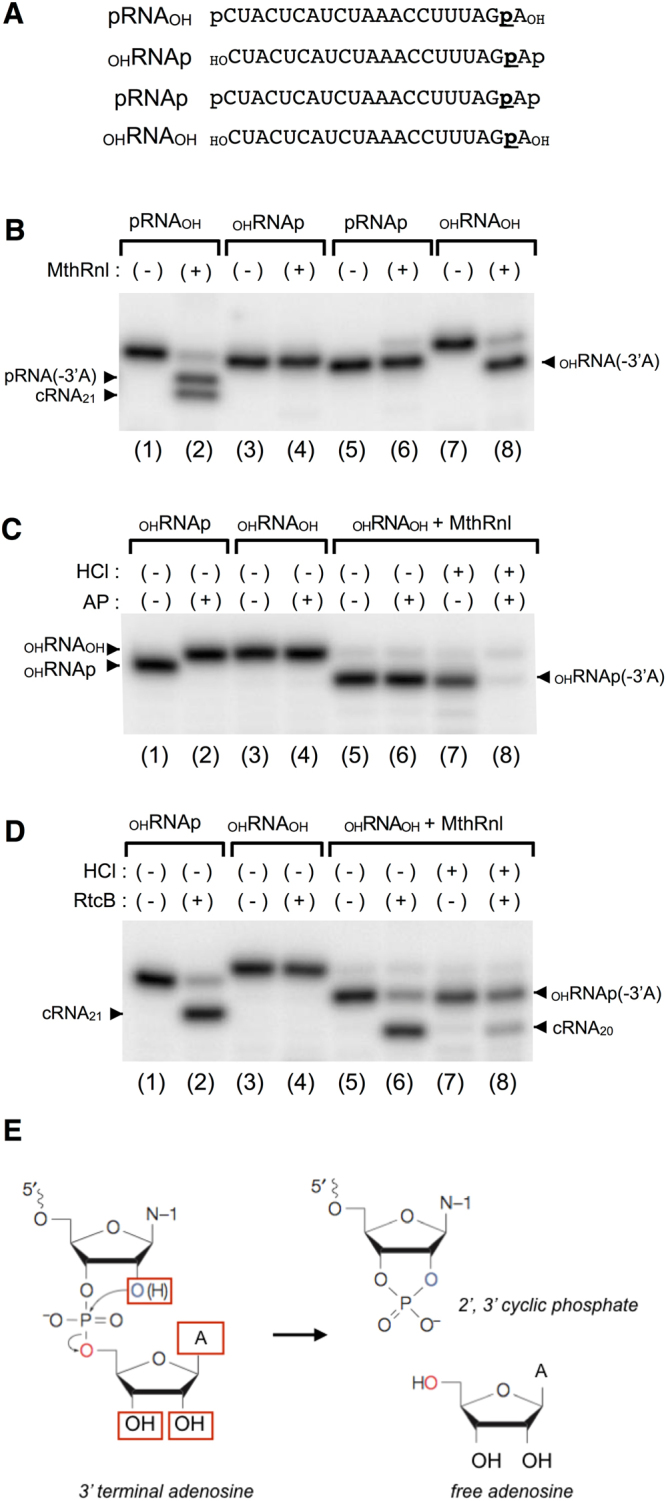
The 3′-end of deadenylated RNA is a 2′,3′-cyclic phosphate group. (**A**) Radiolabeled 21-mer RNAs used in experiments. 21-mer with 5′-OH and 3′-PO_4_ (_OH_RNAp) was prepared by ligating [α-^32^P]pAp to 3′-end of 20-mer synthetic RNA (Supplementary Fig. S5). Other RNAs (_OH_RNA_OH_, pRNA_OH_ and pRNAp) were prepared from radiolabeled _OH_RNAp by enzymatic modification. Position of radiolabled phosphate is underlined. (**B**) 5′-phosphate is not required for RNA 3′-deadenylation. Standard ligation reaction mixture (20 µl) containing 0.45 µg of MthRnl with 1 pmol of either pRNA_OH_, _OH_RNAp, pRNAp, or _OH_RNA_OH_. MthRnl was omitted from a control reaction (-). Positions of 20-mer pRNA(-3′A), _OH_RNA(-3′A) and cRNA (cRNA_20_) are indicated. (**C**) 2′,3′-cyclic phosphate is present at the 3′-end of deadenylated RNA. The 3′-deadenylated _OH_RNA_OH_ was generated by MthRnl and was purified by PAGE (lane 5). Purified 3′-deadenylated _OH_RNA_OH_ (_OH_RNA_OH + _MthRnl) was treated with 0.1 M HCl for 17 hrs at 4 °C, recovered by ethanol precipitation, and then incubated with or without AP (lanes 8 and 7, respectively). (**D**) 3′-deadenylated RNA can be circularized by RtcB. Same as (**C**) except that AP was replaced with *E. coli* RtcB (0.2 µg) in a reaction mixture (20 µl) containing 50 mM Tris-HCl (pH 8.0), 2 mM MnCl_2_ and 100 µM GTP. Positions of 21-mer cRNA (cRNA_21_), 20-mer cRNA (cRNA_20_), and a 20-mer deadenylated _OH_RNA_OH_ [_OH_RNAp(-3′A)] are indicated. (**E**) Proposed mechanism of 3′-deadenylation by archaeal ATP-dependent RNA ligase. Functional groups on the RNA that are required for the 3′-deadenylation reaction are boxed in red. See text for details.

To determine whether the 5′-PO_4_ is required for 3′-deadenylation by MthRnl, we incorporated radiolabeled phosphate into the RNA between the 3′-terminal adenosine and its penultimate nucleoside by ligating [α-^32^P]pAp with T4 Rnl1 (Supplementary Fig. S5). The end of the _OH_RNAp was then enzymatically modified to produce three RNA substrates: one with a 5′-PO_4_ (pRNA_OH_); one with both 5′- and 3′-PO_4_ (pRNAp); and one without a phosphate group (_OH_RNA_OH_) (Fig. 6A and Supplementary Fig. S5). MthRnl was unable to deadenylate or circularize the RNA containing 3′-PO_4_ (Fig. 6B; lanes 4 and 6). However, it efficiently deadenylated 3′-adenosine from _OH_RNA_OH_, as evident from the presence of an RNA species whose migration suggested a 1-nt difference (Fig. 6B; lane 8). We conclude that the 3′-deadenylation reaction is not affected by the 5′-end of the RNA.

### The 3′-terminus of the RNA deadenylated by MthRn1 is a 2′,3′-cyclic phosphate

Theoretically, the 3′-end of pRNA(-3′A) could be occupied by either a 2′,3′cyclic phosphate, a 2′-PO_4_ or a 3′-PO_4_. To distinguish between these possibilities, we isolated the _OH_RNA(-3′A) product generated by MthRnl (Fig. 6B, lane 8) and incubated it with alkaline phosphatase (Fig. 6C, lane 6). This treatment did not alter the mobility or liberate a radiolabeled phosphate from _OH_RNA(-3′A). Treatment of _OH_RNA(-3′A) with acid, which converts the 2′,3′-cyclic phosphate into 2′-PO_4_ and 3′-PO_4_, sensitizes _OH_RNA(-3′A) to alkaline phosphatase (Fig. 6C, lane 8). Thus, _OH_RNA(-3′A) likely possesses a 2′,3′-cyclic phosphate group at its 3′-end. Furthermore, we show that _OH_RNA(-3′A) can be ligated by RtcB, which can join 5′-OH terminated RNA with either 2′,3′-cyclic phosphate or a 3′-PO_4_ end, to form a 20-mer circular RNA (Fig. 6D, lane 6). Pre-treatment of _OH_RNA(-3′A) with HCl inhibits circularization by ~50%; this likely reflects a conversion of 2′,3′-cyclic phosphate into ligatable 3′-PO_4_ and unligatable 2′-PO_4_ ends (Fig. 6D, lane 8). We conclude that the 3′-end of the deadenylated RNA consists of 2′,3′-cyclic phosphate.

## Discussion

During the course of this study to determine the optimal RNA substrate for MthRnl ligation activity, we discovered that MthRnl possesses a 3′-deadenylation activity that selectively cleaves an adenosine residue from the 3′-terminus of an RNA, to yield a 2′,3′-cyclic phosphate end. The 3′-deadenylation activity is specific to RNA, as it requires two ribonucleotides at the 3′-OH end, whereas the rest of the nucleotides can be replaced by DNA. Conversion of the 3′-OH end to a cyclic phosphate prevents ligation to the 5′-PO_4_ end. Once the RNA is 3′-deadenylated, subsequent deadenylation is inhibited due to the presence of a 2′,3′-cyclic phosphate end. Similar to the ligation activity, the 3′-deadenylase activity requires a divalent cation, and excess ATP inhibits the reaction. MthRnl mutant proteins that fail to form the ligase-AMP complex are defective for 3′-deadenylation. In contrast, Thr-117 and Arg-118, which do not affect formation of the ligase-AMP complex (step 1) but are required for formation of the phosphodiester bond (step 3), did not affect the 3′-deadenylation activity^30^. The fact that ATP can inhibit the 3′-deadenylation reaction suggests that the 3′-adenosine on the RNA may compete for the same catalytic site for binding of the ATP. We propose that 3′-adenosine is recognized by the ATP binding pocket in the MthRnl and catalyzes the deadenylation through a transesterification reaction, by stimulating the penultimate ribose 2′-OH to attack the adjacent 3′-5′ phosphodiester to form a 2′,3′-cyclic phosphate and releasing adenosine (Fig. 6E). The active site Lys-97 is not likely involved in transfer of AMP to the 3′-OH end of the substrate, because 3′-adenylated intermediate, pRNA(+pA), was not detected in the reaction, and traced amounts of deadenylated RNA can be detected by K97A mutant protein. Lys-97 may stabilize and/or neutralize the charge on the phosphate between the 3′-adenosine and penultimate nucleoside. The role of divalent cation for 3′-deadenlyation is not clear. The divalent cation does not appear to be required for substrate binding and overall protein folding (Supplemental Fig. S6), or activating the water molecule as a nucleophile, but it may be important for coordinating the active site conformation. With respect to formation of a 2′,3′-cyclic phosphate by removal of a specific nucleoside from the 3′-end, MthRnl appears to have properties similar to those of the Usb1/Mpn1 3′-5′ exonuclease, which trims the U6 snRNA tail. However Usb1/Mpn1 is capable of trimming multiple nucleosides from the 3′-end of the phosphate^8^. Consistent with this difference, the crystal structure of human Usb1 identified that enzyme as a member of the LigT-like superfamily of 2 H phosphoesterases, proteins to which MthRn1 has no structural similarity^7,30^.

Any RNA that terminates with an adenine is a potential substrate for MthRnl 3′-deadenylation. MthRnl efficiently removes 3′-adenosine from RNAs that are single-stranded or have a stem-loop structure with a 3′-single strand overhang. However, it is less efficient in the cases of RNAs that bear a recessed 3′-end, consistent with the notion that the 2′-OH on the penultimate nucleoside needs to be exposed to allow nucleophilic attack of the adjacent adenylate. We also note that the 5′-adenylation activity of MthRnl was generally efficient in the context of a stem-loop RNA bearing a 5′-overhang, but not in the case of one with a recessed 5′-end. The rates of 5′-adenylation and 3′-deadenylation were comparable in RNAs with both 5′- and 3′-overhangs. These results suggest that MthRnl prefers a substrate with an unpaired /unstacked single-stranded RNA end. Whether MthRnl adenylates the 5′-PO_4_ end for ligation or cleaves the 3′-adenosine likely depends on the accessibility of each RNA terminus.

The present study raises the interesting prospect that RNA ligation might not be the only biochemical pathway for MthRnl. We speculate that MthRnl acts on a specific set of 3′-adenylated RNAs to regulate their processing and downstream biological events. RNAs that are exclusively terminated by 3′-adenosine include tRNAs and mRNAs. In the final step of tRNA maturation, the 3′-end is modified by the addition of a -CCA_OH_ group, to which the amino acid is attached. The -CCA sequence protrudes from the acceptor stem helical structure of the tRNA as a single-stranded motif, and is thus a potential substrate for MthRnl 3′-deadenylation. MthRnl may regulate the amount of charged and uncharged tRNA in the cell, as removal of 3′-adenosine and formation of the cyclic end is expected to prevent aminoacylation. Intriguingly, analysis of tRNAs in humans identified a subset that lack 3′-adenosine and terminate in a 2′,3′-cyclic phosphate^33^. This suggests that a 3′-deadenylase activity could also be present in mammalian cells. In some archaea, including methanogens, heteropolymeric poly(A)-rich tails are synthesized and removed by the exosome^34,35^. As in the case of the mammalian U6 snRNA, the formation of 2′,3′-cyclic phosphates could preclude the elongation of poly(A)-rich tails and protect them from degradation machinery from regulating stability of the mRNA. It is also plausible that MthRnl acts as a surveillance enzyme that prevents undesirable intramolecular RNA circularization and intermolecular ligation with the 5′-PO_4_ RNA, by converting the reactive 3′-OH end into a nonreactive 2′,3′-cyclic phosphate. MthRnl may also function in forming a 2′,3′-cyclic phosphate ends that serve as substrates for subsequent ligation with 5′-OH RNA by RtcB for a production of a novel RNA species, or generating an adenylated capped RNA (5′-AppRNA) with a 2′,3′-cyclic phosphate end. In summary, our findings raise possibilities for unexpected mechanisms whereby the MthRn1 could act as a surveillance or editing enzyme, to selectively remove the 3′-adenosine of an RNA to convert the reactive 3′-hydroxyl group into 2′,3′-cyclic phosphate and thereby regulate its metabolism.

## Methods

### RNA substrates

Synthetic RNAs were purchased from Integrated DNA Technologies, Inc. (Coralville, IA, USA). Oligoribonucleotides (1 nmole) were labeled at the 5′-end using 20 units of T4 Pnk in a 20 µl reaction containing 3 nmoles of [γ-^32^P] ATP at 37 °C for 1 hr. Radiolabeled pRNA and pDNA were purified on 13% native polyacrylamide gels.

The 5′-radiolabeled 3′-deadenylated pRNA [pRNA(-3′A)] was prepared from 100 pmol of^32^P-labeled pRNA with a 3′-(5nt) overhang (pUUGUCUGAGAAGACAAGAGA_OH_; prepared using T4 Pnk and [γ-^32^P] ATP as described above) in a reaction mixture (1 ml) containing 50 mM Tris-HCl (pH 6.5), 0.5 mM MgCl_2_, and 90 µg of MthRnl, incubated at 55 °C for 30 min. The reaction was terminated by the addition of 50 µl of 20 mM EDTA, and RNA was extracted with phenol:chloroform:isomylacohol (25:24:1) and precipitated with ethanol. 5′-radiolabeled pRNA(-3′A) was isolated from a 18% polyacrylamide gel by elution at 4 °C for 8 hrs.

### Recombinant RNA ligases

Plasmids that encode wild-type and mutant MthRnl were transformed into *E. coli* BL21(DE3). MthRnl production was induced with IPTG, and His tagged-MthRnl proteins were purified from soluble bacterial extracts by Ni-agarose chromatography as described previously^30^. His tagged-T4 RNA Ligase 1^32^ and T4 RNA Ligase 2^33^ were produced in *E. coli* and purified as described. Protein concentrations were determined with the BioRad dye reagent, using bovine serum albumin (BSA) as the standard.

### Ligation and 3′-deadenylation assay

Standard reaction mixtures (20 µl) containing 50 mM Tris-HCl (pH 6.5), 0.5 mM MgCl_2_, 1 pmol of ^32^P-labeled pRNA and the indicated amount of MthRnl, with or without ATP, were incubated at 55 °C. The reactions were terminated by adding an equal volume of formamide gel loading buffer (90% formamide, 20 mM EDTA). Products were resolved on denaturing 18% (w/v) polyacrylamide (19:1) gels containing 7 M urea in 0.5 × TBE (45 mM Tris borate, 1 mM EDTA). The extent of ligation and 3′-deadenylation were determined by scanning the gel with a Storm Molecular Imager and analyzed by ImageQuant software.

## Electronic supplementary material

Supplementary Figures
