# Supplementary material for: Cleavage of 3′-terminal adenosine by archaeal ATP-dependent RNA ligase
Source: Sci Rep. 2017 Sep 14;7:11662. doi: 10.1038/s41598-017-11693-0 (PMC5599603; doi:10.1038/s41598-017-11693-0)
Supplement: Supplementary file 1 — Supplementary Figures [file 41598_2017_11693_MOESM1_ESM.pdf]

Supplementary Information

Cleavage of 3'-terminal adenosine by archaeal ATP-dependent RNA ligase

Shigeo Yoshinari[1], Yancheng Liu[2], Paul Gollnick[1] and C. Kiong Ho[1, 2, 3]

[1] Department of Biological Sciences, State University of New York, Buffalo, NY 14260, USA
[2] Human Biology Program, School of Integrative and Global Majors, University of Tsukuba, Ibaraki, Japan. 305-8575
[3] Department of Infection Biology, Faculty of Medicine, University of Tsukuba, Ibaraki, Japan. 305-8575

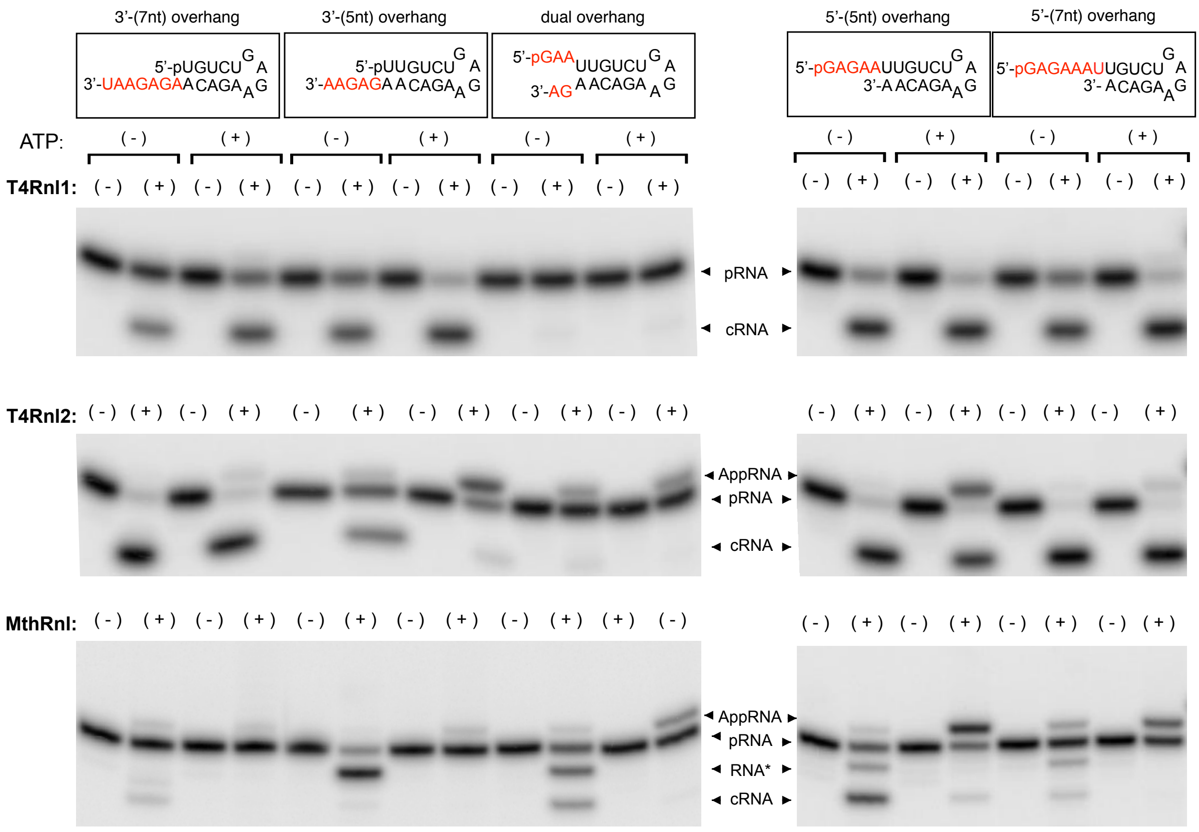

Supplementary Figure S1: The formation of RNA* is specific to archaeal RNA ligase. Bacteriophage RNA ligases do not generate RNA*. *Top panel*: T4 Rnl1 ligation assay. Reaction mixtures (10 μl) contained 50 mM Tris-HCl (pH8.0), 2 mM DTT, 10 mM $MgCl_2$, 1 pmol of indicated ^{32}P-labeled pRNA and 5 pmol T4 Rnl1, with or without 0.2 mM ATP, and were incubated at 37°C for 15 min. *Middle panel*: T4 Rnl2 ligation assay. Reaction mixtures (10 μl) contained 50 mM Tris-HCl (pH 6.5), 5 mM DTT, 5 mM $MgCl_2$, 5 pmol T4 Rnl2 and 1 pmol indicated pRNA substrate, with or without 0.2 mM ATP, and were incubated at 37°C for 15 min. *Bottom panel*: MthRnl ligation assay. Reaction mixtures (10 μl) contained 50 mM Tris-HCl (pH 6.5), 0.5 mM $MgCl_2$, 20 pmol MthRnl and 0.5 pmol pRNA indicated, with or without 1 mM ATP, and were incubated at 55°C for 15 min. The products were resolved by PAGE and visualized using a phosphorimager. Positions of pRNA, AppRNA, cRNA and RNA* are indicated.

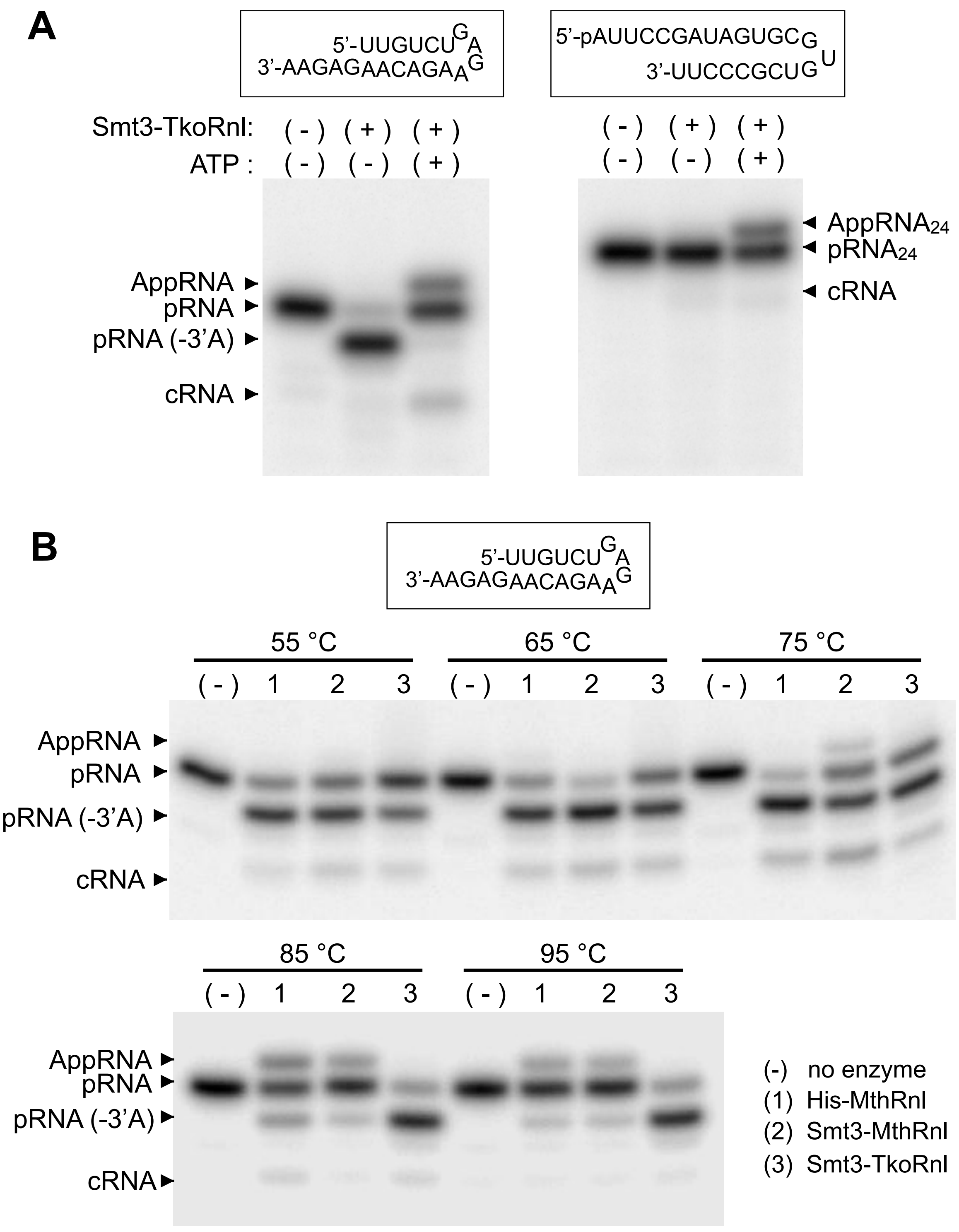

Supplementary Figure S2. T. kodakarensis RNA ligase is capable of generating RNA*. Gene encoding for Thermococcus kodakarensis RNL3 (TK1545) was amplified by PCR and cloned into pET28-smt3 expression vector, which fuses 10-histidine codons and yeast smt3 to the 5' end of TkoRNL. The fusion protein was purified from a soluble bacterial extract by Ni-agarose chromatography. (A) Ligation assay. Reaction mixtures (10 μl) contained 50 mM Tris-HCl (pH7.0), 0.5 mM $MgCl_2$, 0.5 pmol of either 3'-(5nt) over-hang pRNA (left panel) or 24-mer unstructured single-stranded pRNA (right panel), with or without 0.2 mM ATP and 2 μg of Smt3-TkoRnl, were incubated at 85°C for 15 min. (B) Temperature dependency. Reaction mixture contained 3'-(5nt) over-hang pRNA and (1) MthRnl (His-tagged), (2) Smt3-tagged MthRnl or (3) Smt3-TkoRnl, and were incubated at indicated temperature for 15 min. The products were resolved by PAGE and visualized using a phosphorimager. Positions of pRNA, AppRNA, cRNA and pRNA(-3'A) are indicated.

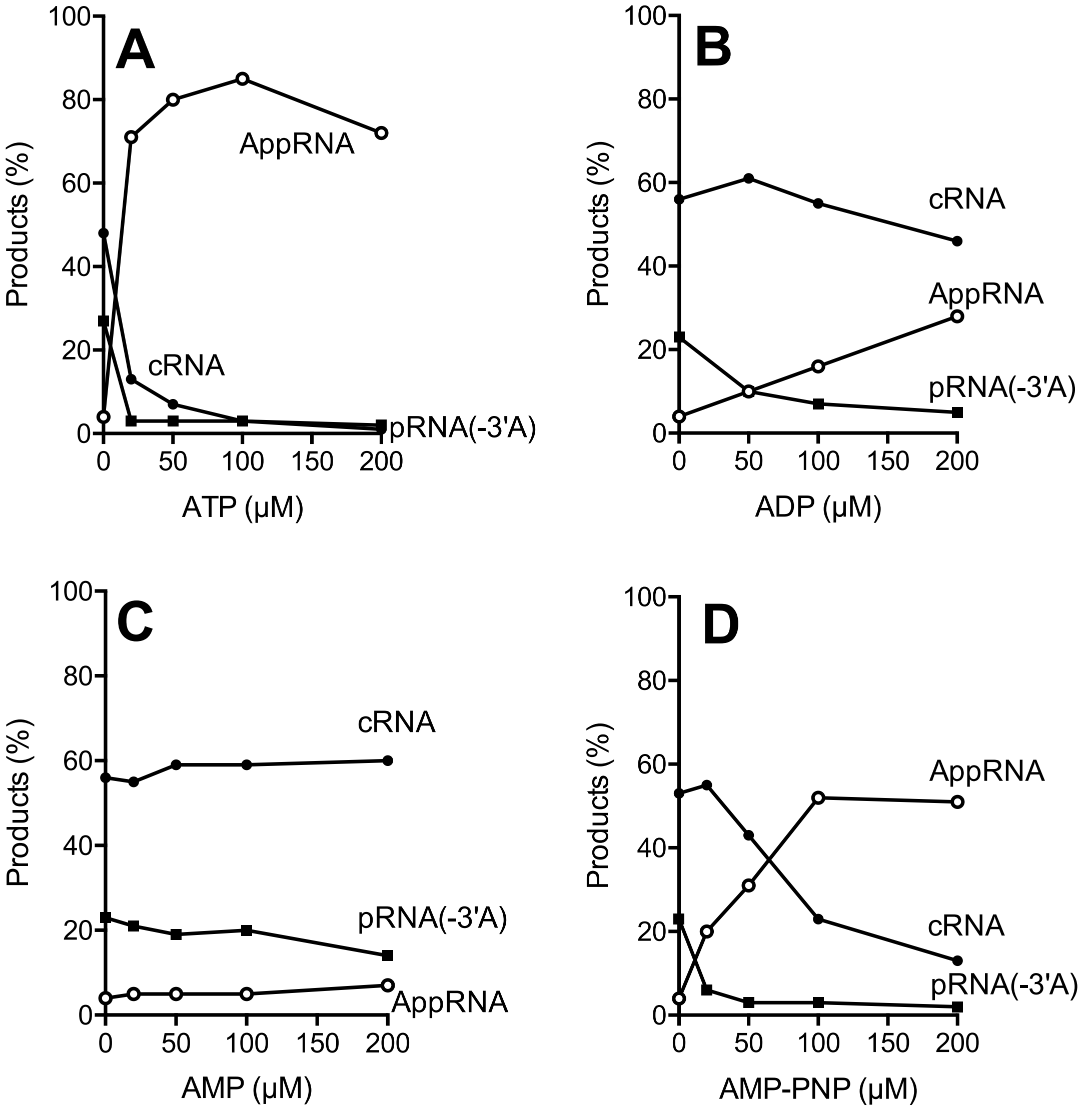

Supplementary Figure S3. Effect of AMP, ADP and AMP-PNP on 3'-deadenylation and RNA circularization. Standard ligation reaction mixture (10*μ*l) containing 1 pmol of ^{32}P-labeled dual overhang pRNA and 10 pmol of MthRnl were incubated with indicated concentration of (A) ATP, (B) ADP, (C) AMP or (D) AMPPNP. Level of cRNA (closed circle), AppRNA (open circle) and pRNA(-3'A) (closed square) formed was plotted as functions of nucleotide or nucleotide analog concentration.

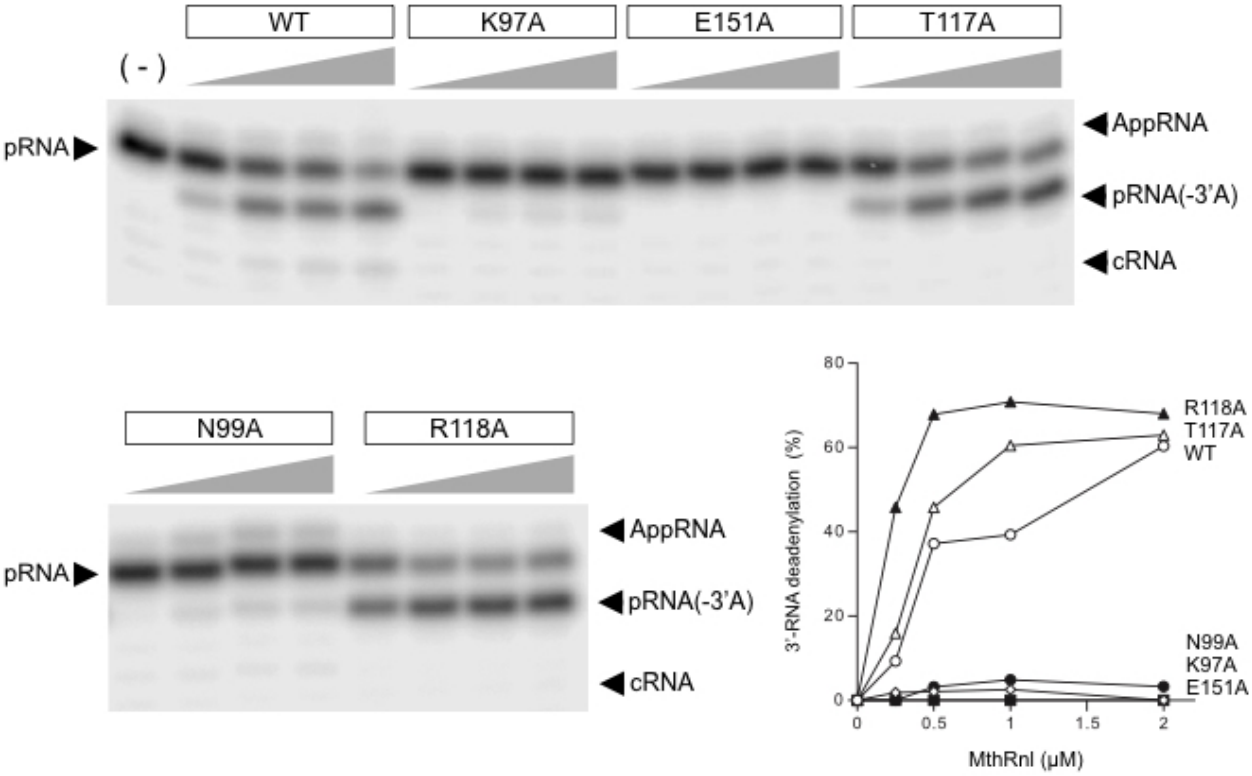

Supplementary Figure S4. Effect of MthRnl mutations on pRNA(-3'A) formation. Standard ligation reaction mixture containing 1 pmol of 3'-(5nt) overhang pRNA and 0.23, 0.45, 0.90 or 1.8 μg (from left to right in each titration series) of wild-type (WT) or mutant MthRnl, as indicated. The products were resolved by PAGE and visualized using a phosphorImager. Positions of pRNA, AppRNA, cRNA and RNA(-3'A) are indicated. *Bottom right:* Percent 3'-RNA deadenylation is plotted as a function of protein concentration.

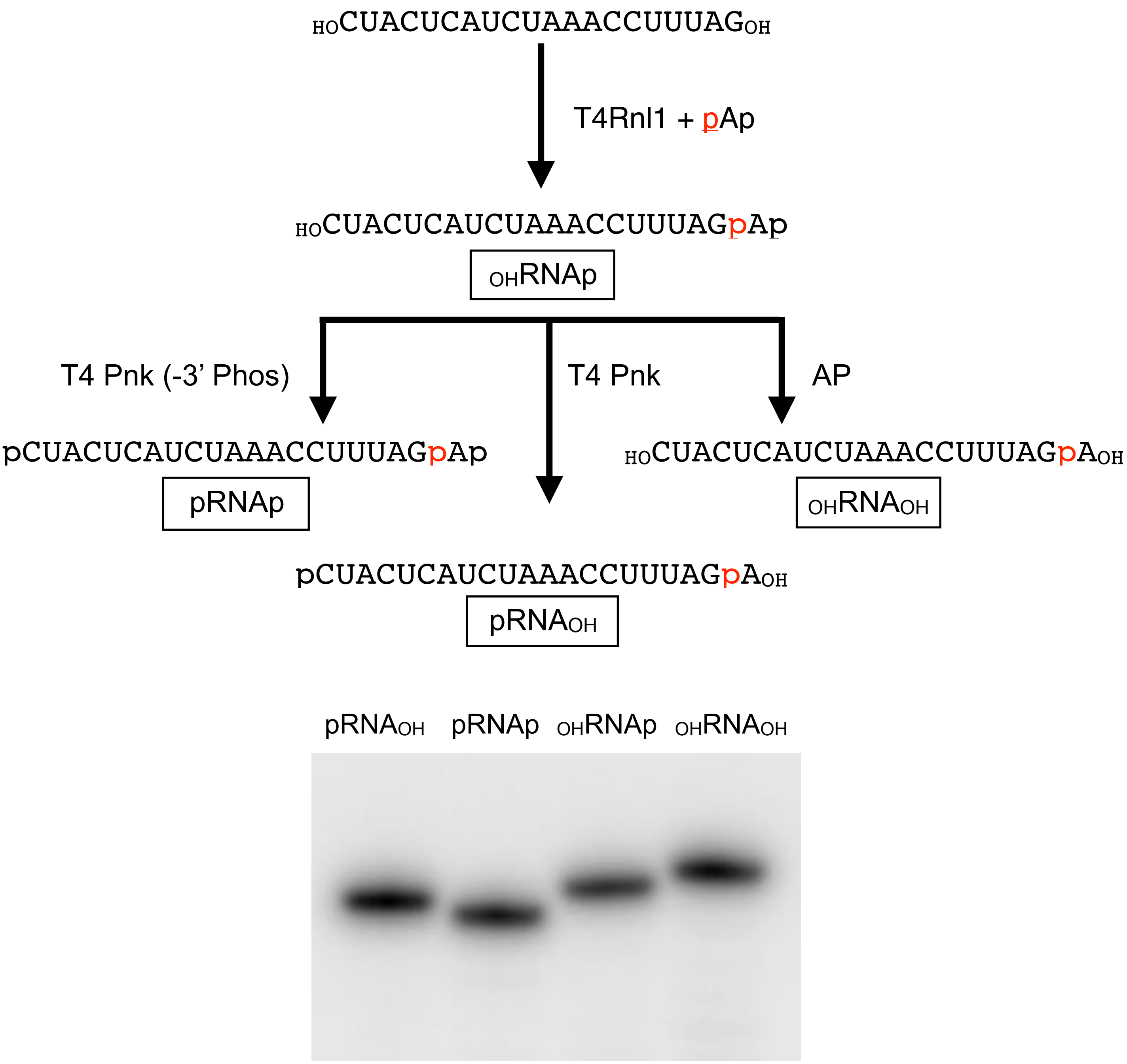

Supplementary Figure S5. Preparation of internally labeled 21-mer RNA. The 3’-^{32}P RNA ($_{OH}$RNAp) was prepared by ligating 20-mer RNA ($_{HO}$CUACUCAUCUAAACCUUUAG$_{OH}$) with [α-^{32}P]pAp using T4 Rnl1. Positions of labeled phosphates are in red and underlined. For preparation of 5’- and 3’-phosphorylated RNA (pRNAp), 5’-phosphorylated RNA (pRNA$_{OH}$) and a hydroxyl-terminated RNA ($_{OH}$RNA$_{OH}$), the ^{32}P-labeled $_{OH}$RNAp was treated with either T4 Pnk (-3’ Phos) or T4 Pnk to add the 5’-phosphate and alkaline phosphatase, respectively. Radiolabled products were gel purified and aliquots (1 pmol) of each preparation were analyzed on a denaturing PAGE gel.

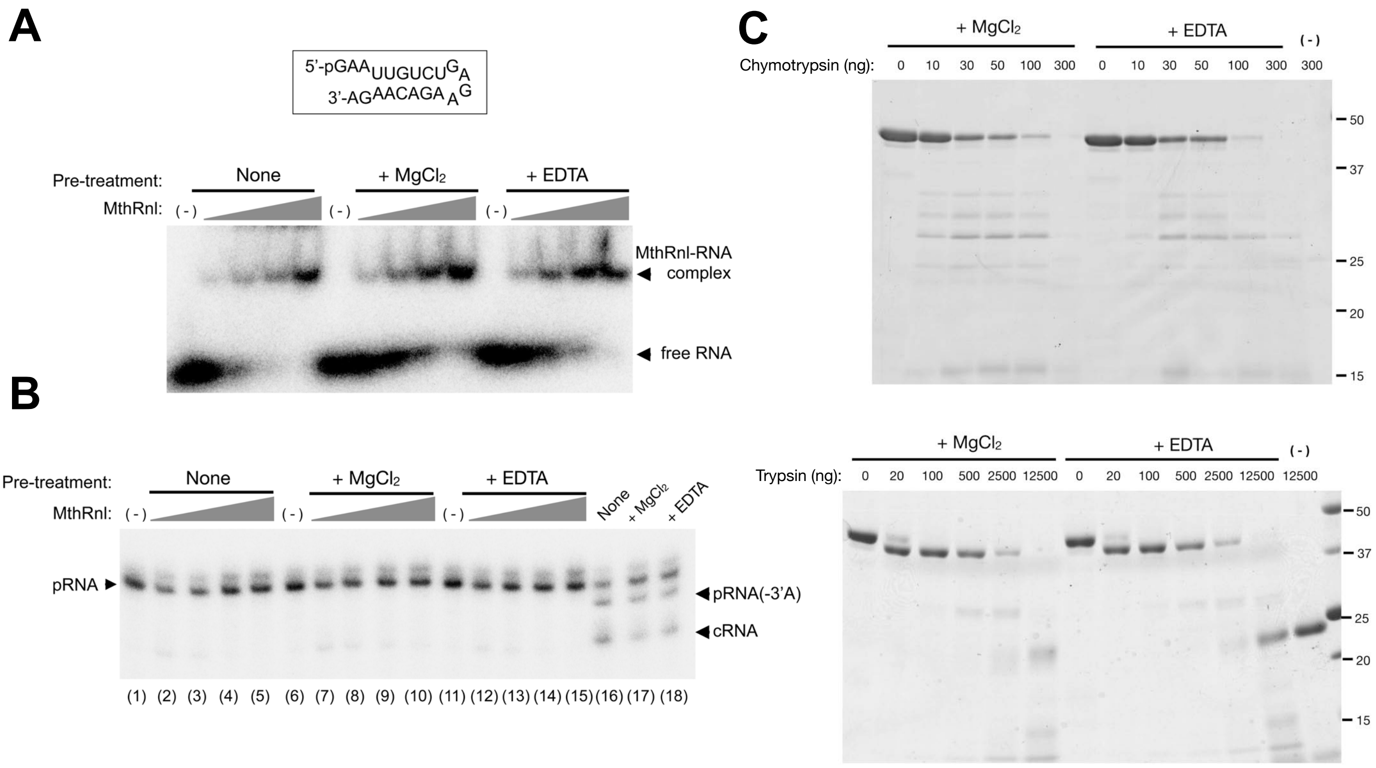

Supplementary Figure S6: Divalent cation does not affect substrate binding or protein folding. MthRnl (100 μg) was incubated with either 10 mM $MgCl_2$ or 2 mM EDTA for 5 min at room temperature, and then applied onto G-50 NICK Column (GE Healthcare) to remove free $MgCl_2$ and EDTA. (A) RNA Binding. Reaction mixture contained 50mM Tris-HCl (pH 6.5), 5% glycerol, 50 nM dual-overhang pRNA indicated with 0.12, 0.23, 0.45, or 0.9 μg (from left to right within each titration series) of MthRnl without treatment (None), with magnesium treatment followed by gel filtration (+$MgCl_2$), or with EDTA treatment followed by gel filtration (+EDTA). MthRnl-RNA complex was visualized by gel mobility shift assay as described (31). (B) RNA Ligation. To verify the integrity MthRnl purified by gel filtration, RNA ligation assay was performed in the presence (lanes 16, 17, 18; 0.9 μg of enzyme) or absence of divalent cation (Lanes 2 -15; with a same enzyme titration series as panel A) using 50 nM dual-overhang pRNA. MthRnl (+$MgCl_2$) or MthRnl (+EDTA) was active for RNA circularization and 3'-deadenylation only when divalent cation is present in the reaction, implies that $MgCl_2$ was successfully removed by gel filtration, and EDTA pre-treatment do not inactivate the enzyme. (C) Limited Proteolytic Digestion. MthRnl (2.5 μg) was incubated with indicated amount of either chymotrypsin (*top panel*) or trypsin (*bottom panel*) in a reaction buffer containing either 1mM $MgCl_2$ or 2 mM EDTA. Incubation was for 15 min at 25°C. Digested products were resolved by 15% SDS-PAGE and stained with Coomassie blue. Control reaction without MthRnl is shown in lane (-). Positions of marker proteins are indicated on the right.
